# SATB2/β-catenin/TCF-LEF pathway induces cellular transformation by generating cancer stem cells in colorectal cancer

**DOI:** 10.1038/s41598-017-05458-y

**Published:** 2017-09-08

**Authors:** Wei Yu, Yiming Ma, Sharmila Shankar, Rakesh K. Srivastava

**Affiliations:** 1 Kansas City VA Medical Center, 4801 Linwood Boulevard, Kansas City, MO 66128 USA; 2 Department of Pathology, University of Missouri-School of Medicine, Kansas City, MO 64108 USA; 3 Department of Pharmaceutical Sciences, University of Missouri-Kansas City, Kansas City, MO 64108 USA; 4 Present Address: Stanley S. Scott Cancer Center, Department of Genetics, Louisiana State University Health Sciences Center, 1700 Tulane Avenue, New Orleans, LA 70112 United States

## Abstract

Recent studies have demonstrated the involvement of colorectal cancer (CRC) stem cells (CSC) in transformation, cancer progression and metastasis. The main goal of this paper was to examine the molecular mechanisms by which SATB2 induced malignant transformation of colorectal epithelial cells. SATB2 induced malignant transformation and these transformed cells gained the characteristics of CSCs by expressing stem cell markers (CD44, CD133, LGR5 and DCLK1) and transcription factors (c-Myc, Nanog and Sox2). Overexpression of SATB2 in normal colorectal epithelial cells increased cell motility, migration and invasion, which were associated with an increase in N-cadherin and Zeb1, and decrease in E-cadherin expression. SATB2 overexpression also upregulated XIAP and cyclin D1, suggesting its role in cell survival and cell cycle. Furthermore, the expression of SATB2 was positively correlated with β-catenin expression in CRC. In contrary, depletion of SATB2 inhibited cell proliferation, colony formation, cell motility and expression of β-catenin, Snail, Slug, Zeb1 and N-cadherin, and upregulated E-cadherin. Furthermore, SATB2 silencing inhibited the expression of stem cell markers, pluripotency maintaining transcription factors, cell cycle and cell proliferation/survival genes and TCF/LEF targets. Finally, β-catenin/TCF-LEF pathway mediated the biological effects of SATB2 in CSCs. These studies support the role of SATB2/β-catenin/TCF-LEF pathway in transformation and carcinogenesis.

## Introduction

Colorectal cancer (CRC) is the third most common malignancy worldwide, and accounts for nearly 1 million newly diagnosed cases and half a million deaths each year^1^. I majority of cases CRC is incurable because of late detection and metastasis^2^. The current clinical treatment mainly includes surgery, chemotherapy, and targeted therapy, but the disease ultimately relapse and is associated with low 5-year survival^3^. There is a significant increase in overall survival for metastatic CRC since the late 1990s coinciding with the introduction and dissemination of new treatment^3, 4^. The colon cancer initiation, progression and metastasis are related to many factors such as genetics, lifestyle, and environmental pollution^4–7^. Most of the CRC develops through hyperplasia, and adenoma. Mounting evidence exists to suggest that CSCs are capable of inducing malignant transformation leading to cancer progression and metastasis^8–11^. Since there are no reliable biomarkers for detection of colon cancer, the management of the disease becomes very difficult. Therefore, improved understanding of the molecular mechanisms underlying CRC carcinogenesis are urgently needed.

SATB2 (special AT-rich binding protein-2), a transcription factor and epigenetic regulator^12, 13^, influences gene expression both by modulating chromatin architecture and by functioning as a transcriptional co-factor^12, 14–17^. SATB2 gene is conserved in humans and mouse. In humans, there are three transcripts which encodes for SATB2 protein. *SATB2* ^−/−^ mice are defective in bone development and osteoblast differentiation^15^. SATB2 is linked to craniofacial patterning and osteoblast differentiation^15^, and in development of cortical neurons^12, 16–18^. SATB2 is over expressed in 85% of CRC tumors, suggesting its use as a diagnostic marker for colon cancer^19^. The Cancer Genome Atlas (TCGA) data confirmed the overexpression of SATB2 gene in CRC^20^. In breast cancer, SATB2 mRNA expression is significantly associated with increasing tumor grade and poorer survival^21^. However, the tumor initiating, promoting and metastatic roles of SATB2 in colorectal carcinogenesis have never been examined.

The pluripotency maintaining factors (Nanog, Oct4, c-Myc, Sox2 and Klf4) regulate self-renewal and survival of stem cells. By promoter analysis, we have identified the SATB2 binding sites in the promoter regions of Nanog, Oct4, SOX-2 and Klf-4, which suggest that SATB2 can act as a “master regulator” of pluripotency in CSCs. Based on these analysis it appears that SATB2 can also serve as an oncogene to promote colon carcinogenesis. However, the clinicopathological significance of SATB2, and its possible mechanism in CRC tumorigenesis and progression is still unclear. Since SATB2 is not expressed in human normal colon epithelial cells, but highly expressed in transformed cells, CSCs and CRC cell lines, it can be used as a diagnostic biomarker for CRC.

During embryonic development Wnt/β-catenin signaling pathway plays a crucial role in regulating cell proliferation and differentiation, whereas in adults it regulates tissue homeostasis and injury repair through generation of stem cells^22–24^. Wnt ligands activate signaling pathway leading to β-catenin stabilization, nuclear translocation, TCF/LEF transcription and induction of β-catenin/TCF target genes^25, 26^. The pathway is also activated by loss or mutations of certain genes. Loss of function of the tumor suppressors APC or Axin2 lead to accumulation of nuclear β-catenin, resulting in the formation of intestinal adenomas^27–29^. Oncogenic point mutations in β-catenin that prevent its degradation also activate this pathway with similar outcomes^28, 30^. Expression of Wnt inhibitor Dickkopf-1 (DKK1)^31, 32^ or deletion of genes encoding β-catenin or Tcf4 blocks crypt proliferation^33^. Some of the targets of TCF/LEF includes pluripotency maintaining factors (c-Myc, Sox-2, Oct-4, Nanog), stem cell marker (CD44), cell cycle and cell survival genes (Cyclin D1 and Survivin), EMT- and metastasis-related genes (Twist, E-cadherin, MMP2, MMP7 and MMP9), and angiogenesis regulator (VEGF)^34^. However, the regulation Wnt/β-catenin signaling pathway by SATB2 has not been examined.

The roles of SATB2 in colon cancer initiation, progression and metastasis are poorly understood. The main goal of the paper is to examine the molecular mechanisms by which SATB2 induces malignant transformation and promotes CRC carcinogenesis. Our data demonstrate that SATB2 is highly expressed in colon cancer cell lines, and primary CRC tissues, and but not in human normal colon epithelial cells and human normal colon, (ii) overexpression of SATB2 can transform human normal colon epithelial cells to progenitor-like cells/CSCs, (iii) inhibition of SATB2 by shRNA attenuates CRC cell proliferation, colony formation and EMT, and (iv) SATB2 expression correlates with nuclear β-catenin expression, and silencing of SATB2 inhibits TCF/LEF activity and its targets. These data suggest that SATB2 can transform normal colon epithelial cells to CSCs/progenitor-like cells which play significant roles in cancer initiation, promotion and metastasis.

## Experimental Procedures

### Cell culture conditions and reagents

Colorectal cancer Colon-320, HT-29, HCT-116, and human normal colon epithelial cells (CRL-1831) were purchased from American Type Culture Collection (ATCC), Manassas, VA. Colon cancer cell lines were grown in Dulbecco’s Modified Eagle’s Medium with 10% Fetal Bovine Serum with antibiotics. The CSCs were isolated from primary tumors and grown in well-defined cell culture medium from Celprogen (Torrance, CA).

Antibodies against CD44, CD133, Nanog, Oct4, Sox2, c-Myc, Bcl-2, Survivin, XIAP, Cyclin D1, E-cadherin, N-cadherin, Snail, Slug, Zeb1, and β-catenin were purchased from Cell Signaling Technology (Danvers, MA). Antibodies against SATB2 and β-actin were purchased from Abcam (Cambridge, MA). Enhanced chemiluminescence (ECL) Western blot detection reagents were purchased from Amersham Life Sciences Inc. (Arlington Heights, IL). A set of GIPZ lentiviral shRNA plasmids against human SATB2 and scrambled shRNA plasmids were purchased from Open Biosystems (Lafayette, CO). pLKO.1 puro shRNA β-catenin plasmid was from Addgene. T-cell factor/lymphoid enhancer factor (TCF/LEF)-responsive pGreenFire Lenti-Reporter construct was purchased from SBI System Biosciences (Palo Alto, CA). SATB2 cDNA was PCR amplified from human cDNA and cloned into NheI/EcoRI digested pSicoR (Addgene). Matrigel was purchased from BD Bioscience (San Jose, CA). Crystal violet was purchased from Sigma-Aldrich (St. Louis, MO). TRIZOL was purchased from Invitrogen (Grand Island, NY). Luciferase assay kit was purchased from Promega (Madison, WI).

### Lentiviral particle production and transduction

The protocol for lentivirus production and transduction have been described elsewhere^35, 36^. In brief, lentivirus was produced by triple transfection of HEK 293 T cells. Packaging 293 T cells were plated in 10-cm plates at a cell density of 5 × 10^6^ a day prior to transfection in DMEM containing 10% heat-inactivated fetal bovine serum. 293 T cells were transfected with 4 µg of plasmid and 4 µg of lentiviral vector using lipid transfection (Lipofectamine-2000/Plus reagent, Invitrogen) according to the manufacturer’s protocol. Viral supernatants were collected and concentrated by adding PEG-it virus precipitation solution (SBI System Biosciences) to produce virus stocks with titers of 1 × 10^8^ to 1 × 10^9^ infectious units per ml. Viral supernatant was collected for three days by ultracentrifugation and concentrated 100-fold. Titers were determined on 293 T cells. Cells were transduced with lentiviral particles expressing gene of interest.

### Motility assay

We used scratch motility assay to monitor the horizontal movement of cells as described elsewhere^37^.

### Transwell migration assay

Transwell migration assay was performed as described elsewhere^35^.

### Transwell invasion assay

Transwell invasion assay was performed as described elsewhere^35^.

### Western blot analysis

The western blot analysis was performed as we described earlier^38^. In brief, cell lysates were subjected to SDS-PAGE, and gels were blotted on nitrocellulose membrane (Amersham Biosciences, Piscataway, NJ, USA). The membranes were blocked with 5% BSA in Tris-Tween buffered saline at 37 °C for 2 h and then incubated with primary antibody diluted in tris-buffered saline (1:1000 dilutions) overnight at 4 °C, with gentle shaking. The membranes were then washed three times with tris-buffered saline-T (TBS-T) and incubated with secondary antibody linked to horseradish peroxidase (1:5000) for 1 h. After incubation with secondary antibody, the membranes were washed again three times with TBS-T. Finally, protein antibody complexes were detected by the addition of ECL substrate (Thermo Fisher Scientific, Rockford, IL).

### Quantitative real-time PCR

Total RNA was isolated using an RNeasy Mini Kit (Qiagen, Valencia, CA). Briefly, cDNA was synthesized using a high capacity cDNA reverse transcription kit (Applied Biosystems). Primers specific for each of the signaling molecules were designed using NCBI/Primer-BLAST and used to generate the PCR products. For the quantification of gene amplification, Real-time PCR was performed using an ABI 7300 Sequence Detection System in the presence of SYBR- Green. The sequence of gene-specific primers are given in supplemental information. Target sequences were amplified at 95 °C for 10 min, followed by 40 cycles of 95 °C for 15 s and 60 °C for 1 min. HK-GAPD was used as endogenous normalization control. All assays were performed in triplicate and were calculated on the basis of ΔΔ*C*t method. The fold change in mRNAs expression was determined according to the method of 2^−ΔΔCT^.

### Immunofluorescence and Immunohistochemistry

For immunofluorescence staining, cells were grown on fibronectin-coated coverslips (Becton Dickinson, Bedford, MA). Cells were fixed with methanol, permeabilized with 1% NP-40, and blocked with 10% BSA, followed by incubating with primary antibody. After washing, cells were incubated with FITC-labeled secondary antibody. Finally, coverslips were washed and mounted using Vectashield (Vector Laboratories, Burlington, CA). Stained slides were examined under a fluorescence microscope. Cells without primary antibody, or with Isotype-specific control IgG, were used as negative controls. Immunohistochemistry of tissues was performed as described elsewhere^35^.

### TCF/LEF reporter assay

Lentiviral particles expressing cop-GFP and luciferase genes (TCF/LEF-mCMV-EF1-Neo) were prepared as described elsewhere^39^. Cells were transduced with lentiviral particles. Transduced cells (5–10,000 cells per well) were seeded in 96-well plates for 48 hrs. At the end of incubation period, luciferase reporter activity was measured as per the manufacturer’s instructions (Promega Corp., Madison, WI).

### Statistical analysis

The mean and SD were calculated for each experimental group with replicates. Differences between groups were analyzed by ANOVA, followed by Bonferoni’s multiple comparison tests using PRISM statistical analysis software (GrafPad Software, Inc., San Diego, CA). Significant differences among groups were calculated at P < 0.05.

## Results

### SATB2 is not expressed in human normal colon epithelial cells, but it is highly expressed in colorectal cancer cell lines

**Figure 1:**
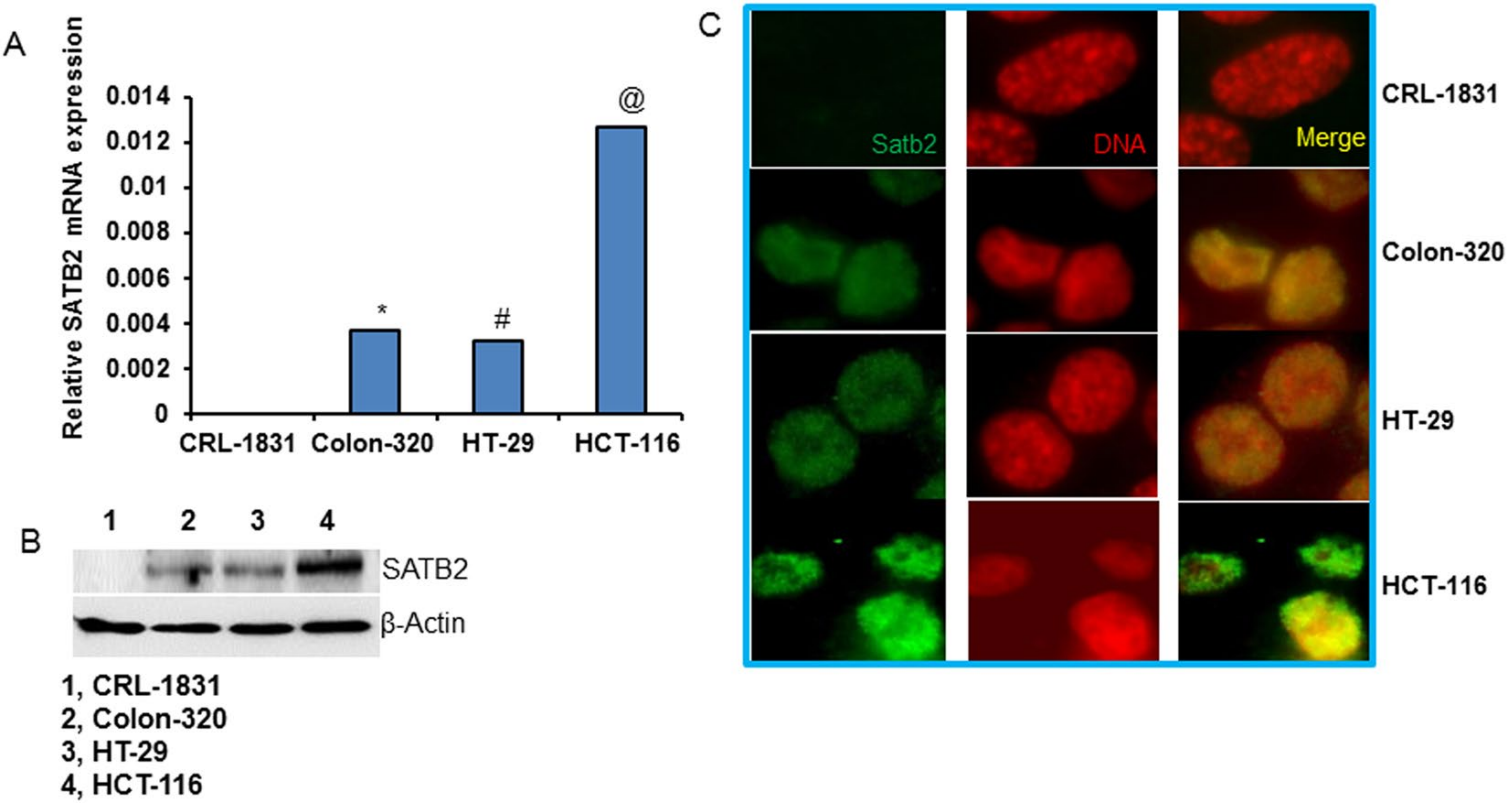
Expression of SATB2 in human normal colon epithelial cells and colorectal cancer cell lines. (**A**) Expression of SATB2 by qRT-PCR. RNA was isolated from human colorectal normal epithelial cells (CRL-1831), and colorectal cancer cell lines (Colon-320, HT-29 and HCT-116), and the expression of SATB2 was measured by qRT-PCR. GAPDH was used as an internal control. Data represent mean (n = 4) ±SD. *, # and @ significantly different from CRL-1831 (P < 0.05). (**B**) Expression of SATB2 protein by the Western blot analysis. Crude proteins from human colorectal normal epithelial cells and colorectal cancer cell lines were isolated and the expression of SATB2 was measured by the Western blot analysis. β-actin was used as a loading control. (**C**) Expression of SATB2 by immunocytochemistry. Colon normal and CRC cell lines were grown on the cover slips and the expression of SATB2 was measured by the immunocytochemistry. IgG was used as a negative control (data not shown).

We first compared the expression of SATB2 in human normal colon epithelial cells (CRL-1831) and colorectal cancer (CRC) cell lines (Colon-320, HT-29 and HCT-116) by qRT-PCR, Western blot analysis and immunocytochemistry (ICC). As shown in Fig. 1A, SATB2 is not expressed in human normal colon epithelial CRL-1831 cells. However, it is highly expressed in CRC Colon-320, HT-29 and HCT-116 cell lines. Interestingly, the expression of SATB2 was highest in HCT-116 cell line.

We next confirmed the expression of SATB2 by the Western blot analysis and ICC. As shown in Fig. 1B, SATB2 is not expressed in human normal colon epithelial CRL-1831 cells, whereas the significant expression of SATB2 protein was notices in CRC Colon-320, HT-29 and HCT-116 cell lines. HCT-116 cells expressed the highest level of SATB2 protein. Similar expression pattern of SATB2 was observed by ICC (Fig. 1C). These data suggest that the expression of SATB2 is tightly regulated in CRC.

### Overexpression of SATB2 in human normal colon epithelial (CRL-1831) cells induces cellular transformation and stemness (by expressing stem cell markers and pluripotency maintaining factors)

**Figure 2:**
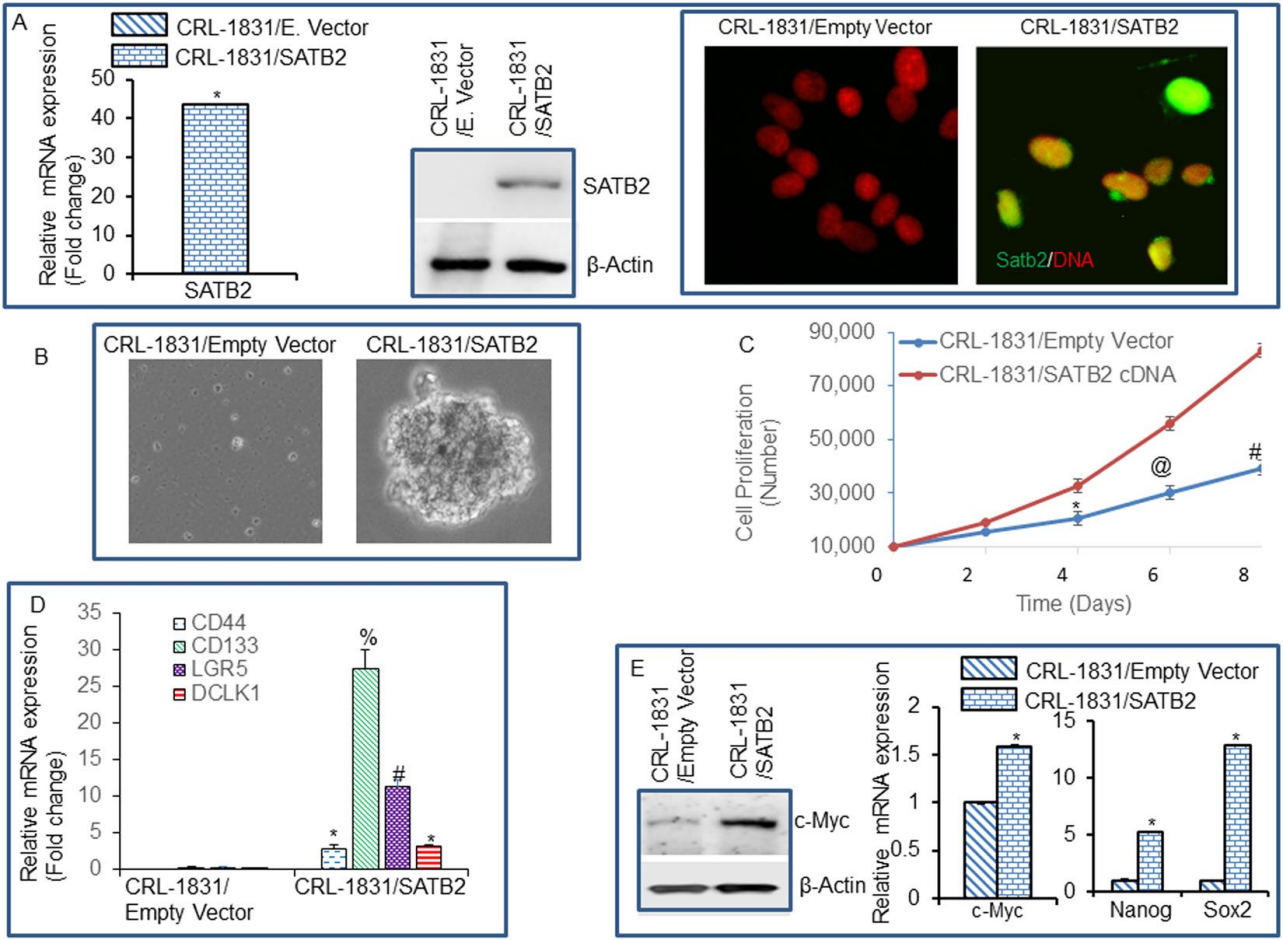
Overexpression of SATB2 in CRL-1831 cells induces cellular transformation and stemness. (**A**) Expression of SATB2. CRL-1831 cells were stably transduced with lentiviral particles expressing either empty vector or SATB2 gene. SATB2 expression was measured the qRT-PCR, Western blot analysis and immunocytochemistry (ICC). (**B**) Spheroid formation in suspension. CRL-1831/Empty Vector and CRL-1831/SATB2 cells were grown in suspension for two week in ultralow attachment plate in a well-defined stem cell medium. Pictures of spheroids in suspension were taken by microscope. (**C**) Proliferation Assay. CRL-1831/Empty Vector and CRL-1831/SATB2 cells were grown and proliferation was measured at day 2, 4, 6 and 8. Data represent mean (n = 4) ±SD. *, @ and # significantly different from respective empty vector group, P < 0.05. (**D**) RNA was isolated and the expression of stem cell markers (CD44, CD133, LGR5 and DCLK1) was measured by qRT-PCR analysis. Data represent mean (n = 4) ±SD. *, %, and # significantly different from CRL-1831/Empty Vector group (P < 0.05). (**E**) Expression of pluripotency maintaining factors. Left Panel, Western blot analysis was performed to measure the expression of c-Myc in CRL-1831/Empty Vector and CRL-1831/SATB2 groups. Right Panel, RNA was isolated and the expression of c-Myc, Nanog and Sox2 was measured by qRT-PCR analysis. Data represent mean (n = 4) ±SD. *Significantly different from CRL-1831/Empty Vector group (P < 0.05). Gene expression of Empty Vector was normalized to 1.

The cell transformation is an early event in carcinogenesis which is characterized by (i) the ability of cells to grow under high saturation density with less oriented conditions, (ii) the loss of contact inhibition and tight junction, and (iii) the ability of cells to form colonies^39, 40^. In order to prove that SATB2 induces cellular transformation and stemness, we overexpressed SATB2 in CRL-1831 cells. Lentiviral mediated infection of SATB2 gene in CRL-1831 (CRL-1831/SATB2) cells resulted in an increased expression of SATB2 mRNA and protein (Fig. 2A). Similarly, ICC data confirmed that the SATB2 was highly expressed in CRL-1831/SATB2 cells (right panel). The expression of SATB2 was not observed in CRL-1831 cells.

We next examined whether SATB2 induces transformation. Overexpression of SATB2 induces cellular transformation as evident by formation of spheroids in suspension (Fig. 2B). Normal CRL-1831 cells (CRL-1831/empty vector) were unable to form spheroids in suspension. Furthermore, CRL-1831/SATB2 cells demonstrated enhanced cell growth compared to CRL-1831/empty vector cells (Fig. 2C). We next measured whether the STAB2-transformed cells gained the properties of stemness by expressing stem cell markers and pluripotency maintaining factor. Overexpression of SATB2 in CRL-1831 cells resulted in induction of stem cell markers (CD44, CD133, LGR5 and DCLK1), and pluripotency maintaining transcription factors (c-Myc, Nanog and Sox2) (Fig. 2D and E). Overall, these data suggest that SATB2 can induce cellular transformation in CRL-1831 cells by inducing CSCs/progenitor cells which are capable of expressing stem cell markers and pluripotency maintaining transcription factors.

### Overexpression of SATB2 in CRL-1831 cells induces epithelial to mesenchymal transition

**Figure 3:**
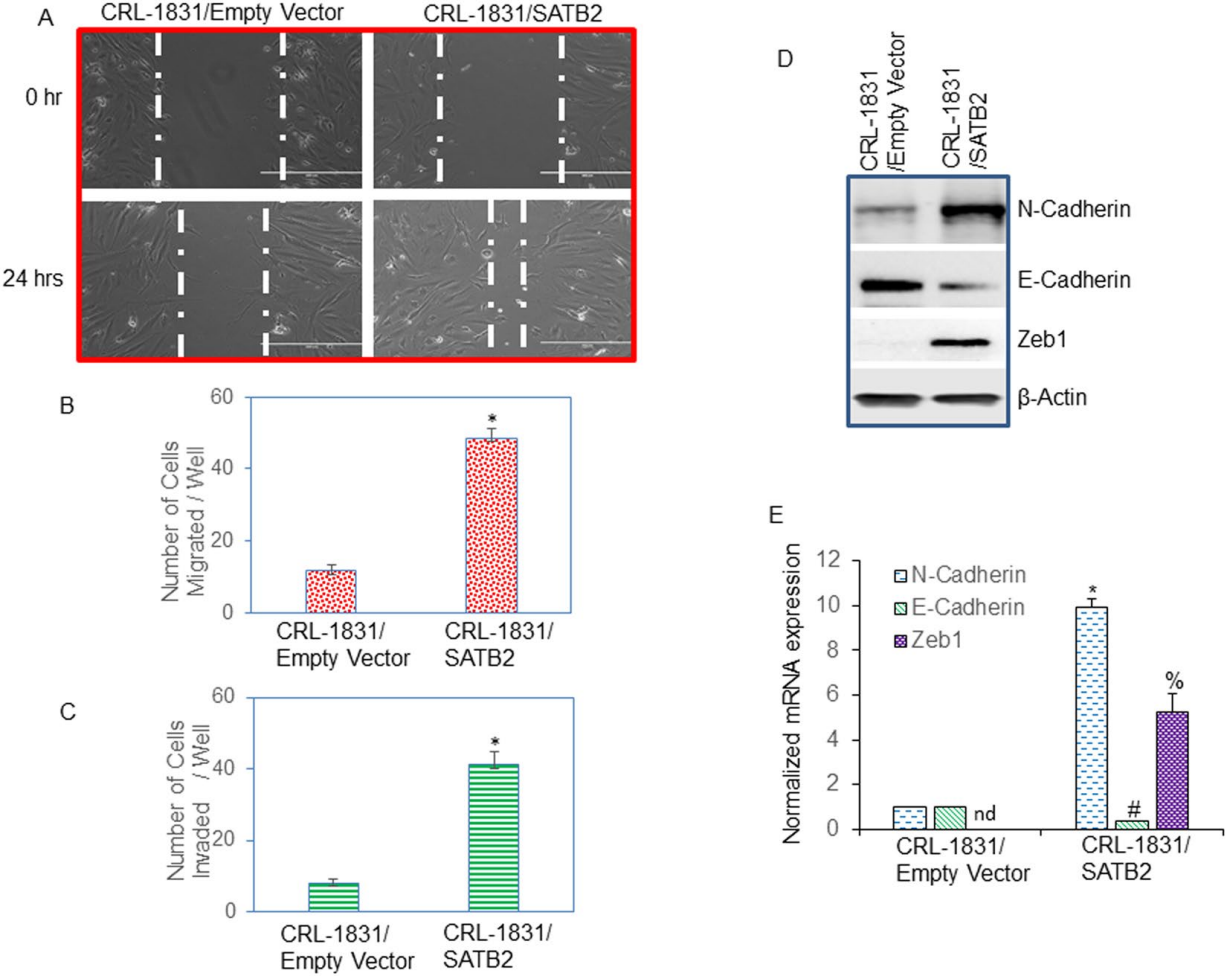
Overexpression of SATB2 in CRL-1831 cells induces EMT characteristics (**A**) Cell Motility assay. CRL-1831/Empty Vector and CRL-1831/SATB2 cDNA cells were grown in petri dishes. After cell attachment, scratch lines were made with the fine pipette tips in both the groups. Phase contrast images of scratched cells were captured at 0 h and 24 h time points. (**B**,**C**) Cell migration and invasion assays. CRL-1831/Empty Vector and CRL-1831/SATB2 cDNA were seeded for 48 h. Cell migration and invasion assays were performed as described in Materials and Methods. Data represent mean (n = 4) ±SD. *Significantly different from control (CRL-1831/Empty Vector) group, P < 0.05. (**D**) Expression of N-Cadherin, E-Cadherin and Zeb1 in CRL-1831/Empty Vector and CRL-1831/SATB2 cDNA groups. The expression of N-Cadherin, E-cadherin and Zeb1 was measured by the Western blot analysis. (**E**) RNA was isolated and the expression of N-Cadherin, E-Cadherin and Zeb1 was measured by qRT-PCR analysis. Data represent mean (n = 4) ±SD. *, #, and % significantly different from CRL-1831/Empty Vector group (P < 0.05). Gene expression of Empty Vector group was normalized to 1. nd = not detected.

Epithelial to mesenchymal transition (EMT) is a process whereby epithelial cells undergo transition to a mesenchymal phenotype and contributes directly to stemness and cancer cell metastasis^41^. We therefore sought to examine whether overexpression of SATB2 in normal CRL-1831 cell enhances cell motility, migration and invasion. As shown in Fig. 3A, overexpression of SATB2 in CRL-1831 cells enhanced cell motility. Similarly, overexpression of SATB2 in CRL-1831 cells enhanced cell migration and invasion compared to CRL-1831/Empty Vector group (Fig. 3B and C).

During EMT, the expression of transcription factors Zeb1 is induced, resulting in regulation of cadherins^41^. We therefore compared the expression of Zeb1 in CRL-1831/Empty Vector and CRL-1831/SATB2 cells. We measured the expression of N-Cadherin, E-Cadherin and Zeb1 by the Western blot analysis and qRT-PCR. Overexpression of SATB2 in CRL-1831 (CRL-1831/SATB2) cells resulted in induction of Zeb1 mRNA and protein compared to that in CRL-1831/Empty Vector cells (Fig. 3D and E). Furthermore, CRL-1831/SATB2 cells demonstrated a higher expression of N-Cadherin and a reduced expression of E-Cadherin at mRNA and protein levels. These data suggest that SATB2 gene is capable of inducing EMT in CRL-1831 cells by inducing EMT-related transcription factor Zeb1 and causing the “cadherin switch”.

### Overexpression of SATB2 in CRL-1831 cells upregulates expression of XIAP and cyclin D1

**Figure 4:**
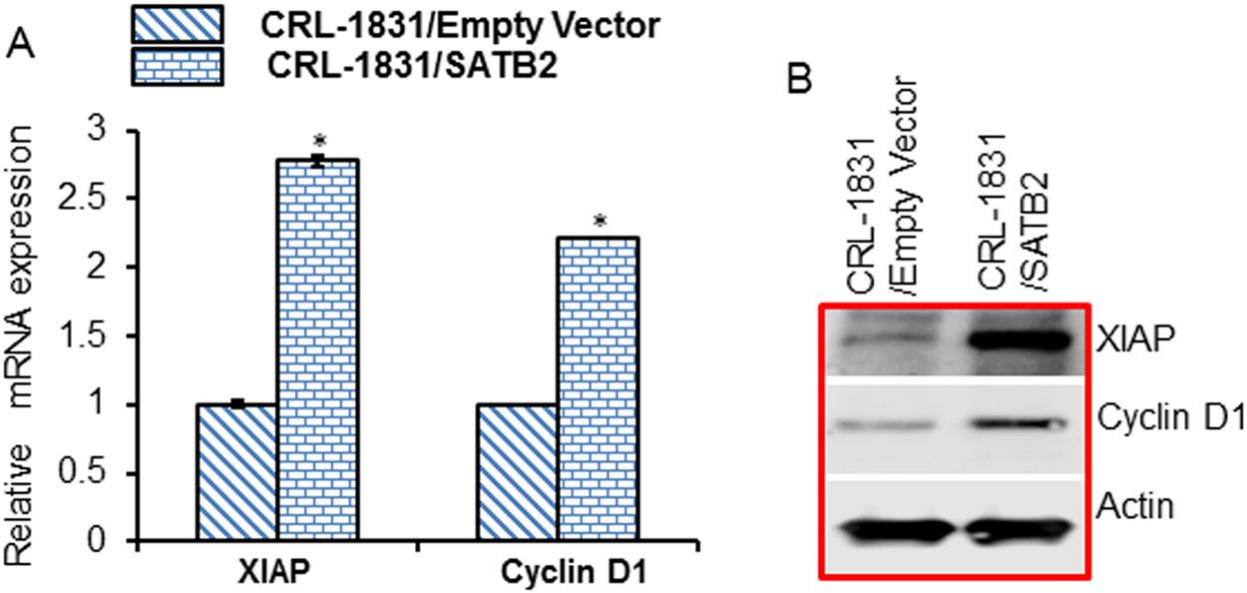
Overexpression of SATB2 in CRL-1831 cells upregulates XIAP and cyclin D1. (**A**) Expression of XIAP and Cyclin D1 in CRL-1831/Empty Vector and CRL-1831/SATB2 cDNA cells. The expression of XIAP and Cyclin D1 was measured by the qRT-PCR. Data represent mean (n = 4) ±SD. (**B**) Expression of XIAP and Cyclin D1 in CRL-1831/Empty Vector and CRL-1831/SATB2 cDNA cells. The expression of XIAP and Cyclin D1 was measured by the Western blot analysis. β-actin was used as a loading control.

Cell proliferation and cell cycle are regulated by XIAP and Cyclin D1, respectively. We therefore compared the expression of XIAP and Cyclin D1 in CRL-1831/Empty Vector and CRL-1831/SATB2 cells. Overexpression of SATB2 resulted in upregulation of XIAP and Cyclin D1 in CRL-1831/SATB2 cells compared to that in CRL-1831/Empty Vector cells (Fig. 4A).

We next confirmed the mRNA data with the Western blot analysis. As shown in Fig. 4B, overexpression of SATB2 resulted in upregulation of XIAP and Cyclin D1 proteins in CRL-1831/SATB2 cells compared to that in CRL-1831/Empty Vector cells (Fig. 4B). These data suggest that SATB-transformed cells may gain enhanced ability to survive and proliferate by modulating the expression of XIAP and Cyclin D1.

### Knockdown of SATB2 in colorectal cancer cell lines inhibits cell proliferation, and colony formation

**Figure 5:**
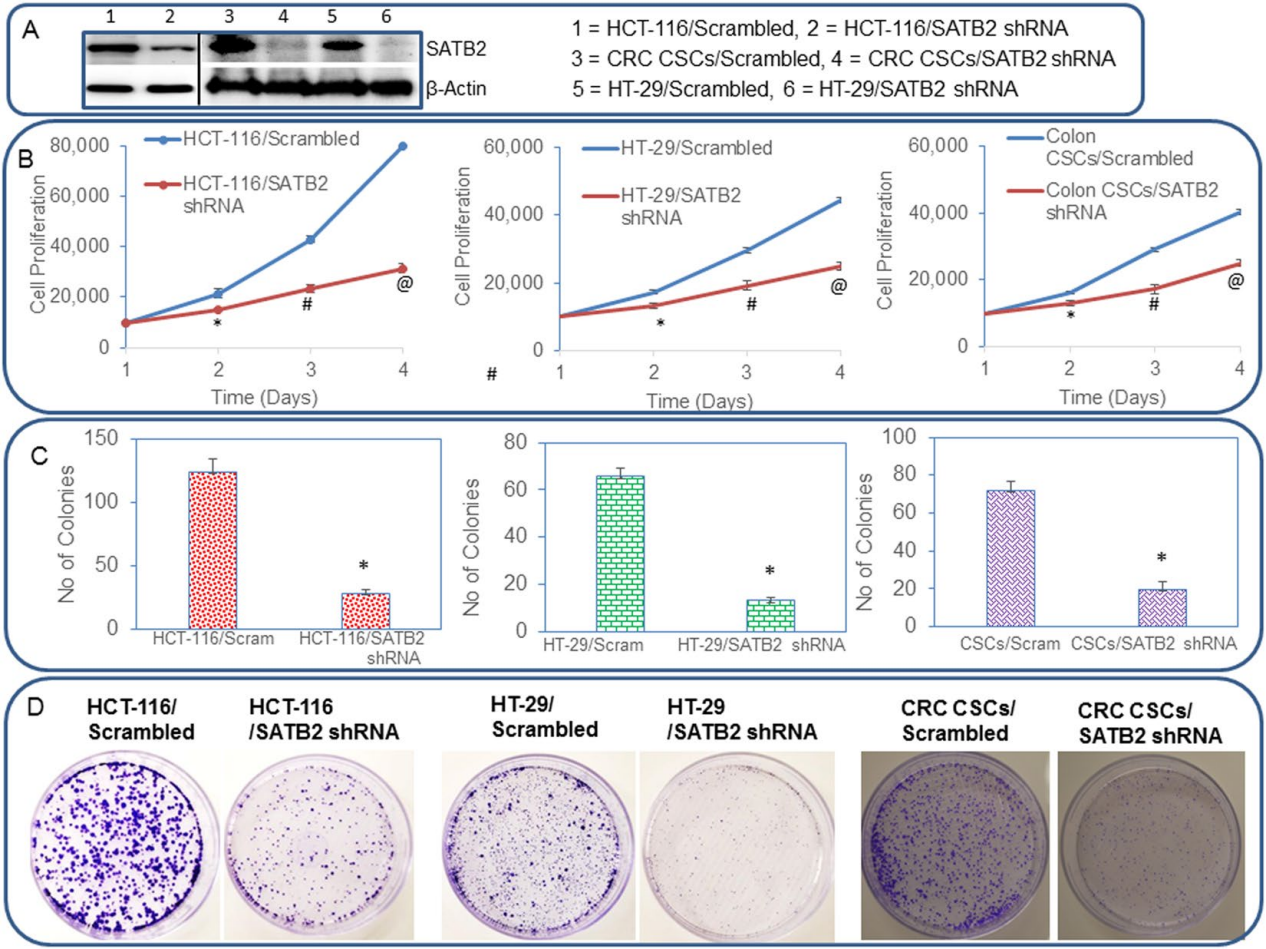
SATB2 shRNA inhibits cell proliferation and colony formation in colorectal cancer HCT-116 and HT-29 cells, and CSCs. (**A**) HCT-116, HT-29 and CSCs were transduced with lentiviral particles expressing either Scrambled or SATB2 shRNA. A validated mixture of constructs which target 4 sites on SATB2 gene was used (Open Biosystems, Pittsburgh PA). Western blot analysis was performed to measure the expression of SATB2. β-actin was used as a loading control. (**B**) Cell proliferation of HCT-116/Scrambled, HCT-116/SATB2 shRNA, HT-29/Scrambled, HT-29/SATB2 shRNA, CSCs/Scrambled, and CSCs/SATB2 shRNA groups was measured over time. Data represent mean (n = 4) ±SD. ^*^, #, @ Significantly different from Scrambled group (P < 0.05). (**C**,**D**) Colony formation Assay. HCT-116/Scrambled, HCT-116/SATB2 shRNA, HT-29/Scrambled, HT-29/SATB2 shRNA, CSCs/Scrambled, and CSCs/SATB2 shRNA cells were seeded, and colonies formed at 21 days were counted and photographed. Data represent mean (n = 4) ±SD. *Significantly different from Scrambled group (P < 0.05).

The transcription factor SATB2 regulates gene expression which participates in stemness, cell survival and differentiation^42–45^. We next examined whether inhibition of SATB2 attenuates cell growth of CRC cells and CSCs. HCT-116 and HT-29 cancer cells and CSCs were transduced with either scrambled or SATB2 shRNA lentiviral particles, and SATB2 expression and cell growth were measured. Knockdown of SATB2 by shRNA inhibited SATB2 expression in HCT-116, HT-29 and CSCs, as measured by the Western blot analysis (Fig. 5A). HCT-116/SATB2 shRNA, HT-29/SATB2 shRNA and CRC CSCs/SATB2 shRNA groups had lower growth rates than HCT-116/Scrambled, HT-29/Scrambled cells and CRC CSCs/Scrambled groups, respectively (Fig. 5B). These data suggest that the knockdown of SATB2 in cancer cells and CSCs can suppress CRC growth.

We next examined the effects of SATB2 on colony formation. The inhibition SATB2 expression by shRNA attenuated colony formation in HCT-116/SATB2 shRNA, HT-29/SATB2 shRNA and CRC CSCs/SATB2 shRNA groups compared to their scrambled control groups (Fig. 5C and D). These data suggest that inhibition of SATB2 expression in colorectal cancer cells and CSCs can retard cell proliferation and colony formation.

### Knockdown of SATB2 in colorectal cancer cell lines inhibits epithelial to mesenchymal transition

**Figure 6:**
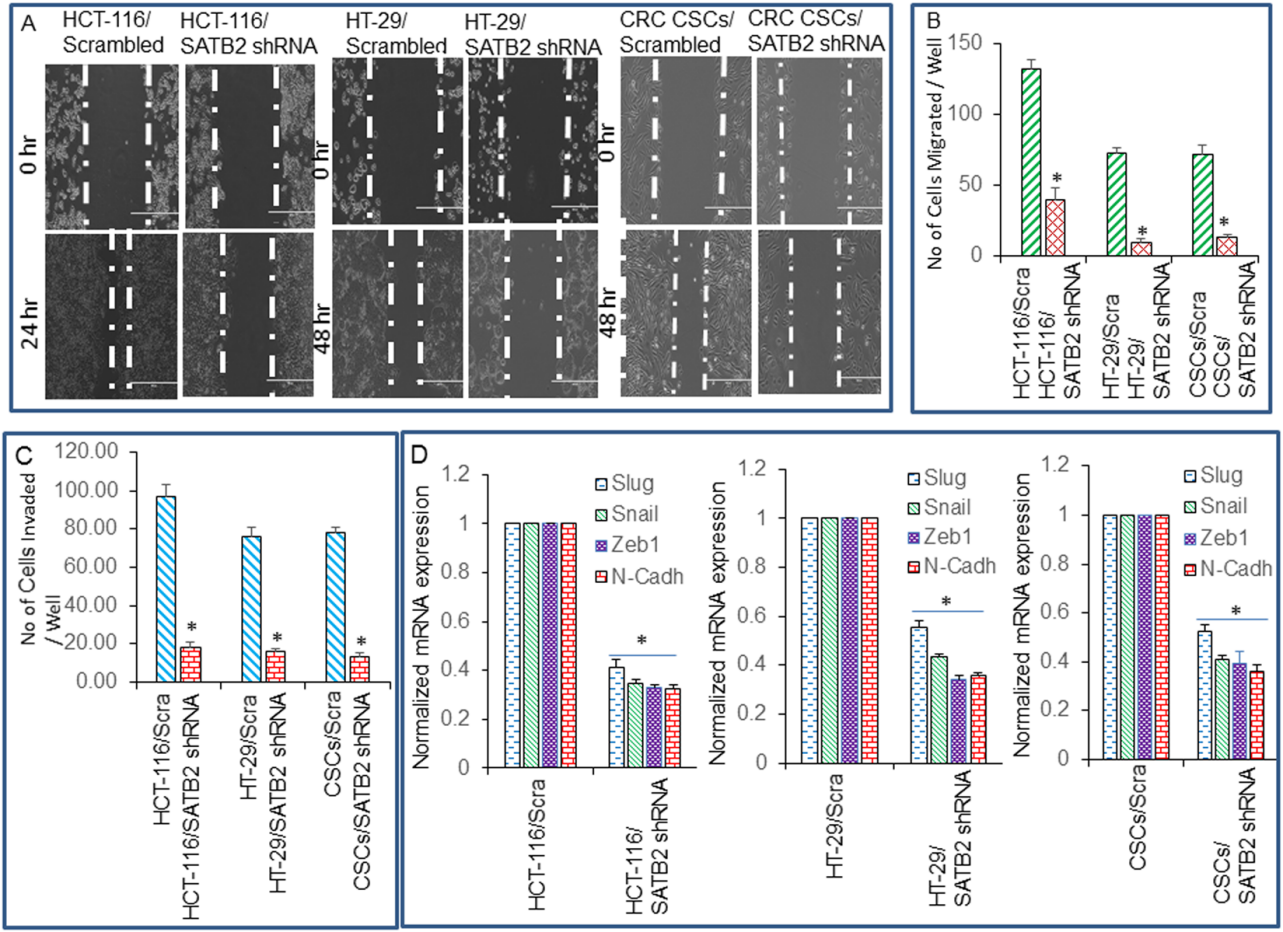
SATB2 shRNA inhibits cell motility, and regulates EMT-related genes in CRC cell lines and CSCs. (**A**) Cell Motility Assay. HCT-116/Scrambled, HCT-116/SATB2 shRNA, HT-29/Scrambled, HT-29/SATB2 shRNA, CSCs/Scrambled, and CSCs/SATB2 shRNA cells were grown in petri dishes. After 18 hours of incubation, cells were scratched with the fine pipette tips. Phase contrast images of scratched cells were captured at 0 h, 24 h or 48 h time points. (**B**,**C**) Cell invasion and migration assays. HCT-116/Scrambled, HCT-116/SATB2 shRNA, HT-29/Scrambled, HT-29/SATB2 shRNA, CSCs/Scrambled, and CSCs/SATB2 shRNA cells were seeded for 48 h. Cell migration and invasion assays were performed as described in Materials and Methods. Data represent mean (n = 4) ±SD. *Significantly different from scrambled (Scra) group, P < 0.05. (**D**) Expression of EMT-related genes. The expression of Slug, Snail, Zeb1 and N-Cadherin in HCT-116/Scrambled, HCT-116/SATB2 shRNA, HT-29/Scrambled, HT-29/SATB2 shRNA, CSCs/Scrambled, and CSCs/SATB2 shRNA cells were measured by qRT-PCR. GAPDH was used as an internal control. Data represent mean (n = 4) ±SD. *Significantly different from scrambled control group (P < 0.05). Cadh = Cadherin.

EMT plays a key role in cancer progression and metastasis^41^. The cells undergoing EMT upregulate the expression of cell motility-related genes and show enhanced migration and invasion. The hallmarks of EMT in malignant cells include changed cell morphology and increased metastatic capabilities in cell migration and invasion^41^. We therefore examined whether inhibition of SATB2 attenuates EMT characteristics in CRC cells and CSCs. HCT-116 and HT-29 cell lines and CSCs were transduced with either scrambled or SATB2 shRNA lentiviral particles as described above. SATB2 shRNA inhibited cell motility of HCT-116 and HT-29 cells and CSCs (Fig. 6A). Similarly, SATB2 shRNA inhibited cell migration and invasion of HCT-116 and HT-29 cell lines, and CSCs (Fig. 6B and C).

We next examined the effects of inhibiting SATB2 on the expression of EMT transcription factors and mesenchymal marker N-cadherin in HCT-116 and HT-29 cell lines and CSCs by qRT-PCR (Fig. 6D). Transduction of HCT-116 and HT-29 cell lines and CSCs with SATB2 shRNA lentiviral particles inhibited the expression of Slug, Snail, Zeb1 and N-Cadherin. These data suggest that inhibition of SATB2 expression in CRC can suppress EMT characteristics in part by inhibiting cell motility, and the expression of EMT-related transcription factors and N-cadherin, and upregulating the expression of N-cadherin.

### Knockdown of SATB2 in colorectal CSCs inhibits makers of stem cells, pluripotency, cell cycle, and cell survival/proliferation, and β-catenin/TCF/LEF pathway

**Figure 7:**
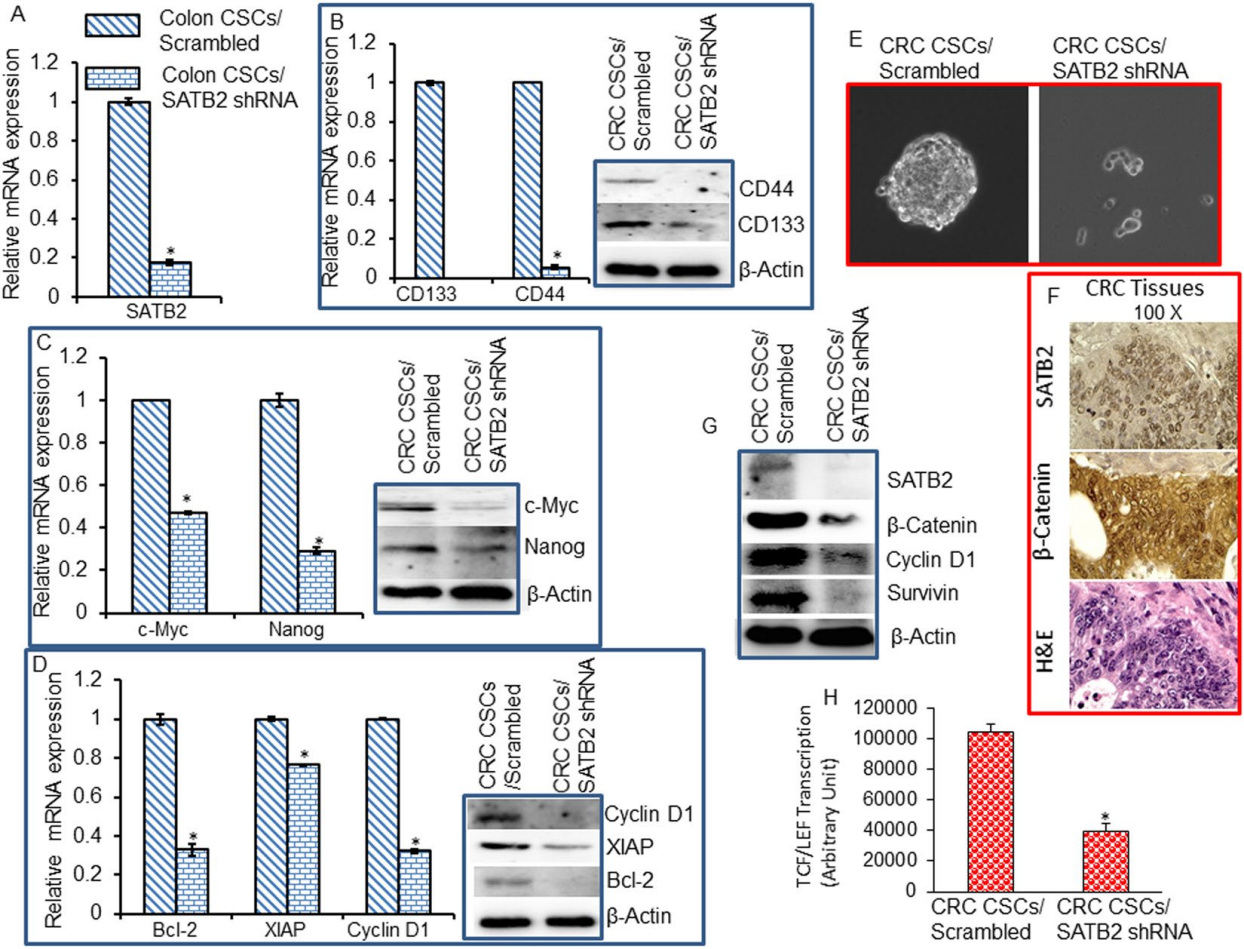
Inhibition of markers of CSCs, pluripotency/self-renewal, cell cycle, cell survival/proliferation by SATB2 shRNA. (**A**) Colorectal CSCs were transduced with lentiviral particles expressing either Scrambled or SATB2 shRNA. SATB2 mRNA expression was measured by qRT-PCR. Data represent mean (n = 4) ±SD. *Significantly different between groups (P < 0.05). (**B**) RNA and protein expression of CSC’s markers. The mRNA expression of CD133 and CD44 was measured by qRT-PCR. Cell lysates from CSC/Scrambled and CSCs/SATB2 shRNA groups were prepared and the expression of CD44, and CD133 was measured by the WB analysis. (**C**) RNA and protein expression of pluripotency maintaining factors. The mRNA expression of c-Myc and Nanog was measured by qRT-PCR. The protein expression of c-Myc and Nanog was measured by the WB analysis. (**D**) RNA and protein expression of Bcl-2, XIAP and Cyclin D1. The mRNA expression of c-Myc and Nanog was measured by qRT-PCR. The protein expression of Cyclin D1, XIAP, and Bcl-2 was measured by the WB analysis. (**E**) Spheroid formation. Cells from CSC/Scrambled and CSCs/SATB2 shRNA groups were seeded in ultralow six-well plate. Spheroids formed after 12 days were photographed. (**F**) Expression of SATB2 and nuclear β-catenin in human CRC tissues was measured by IHC. H&E staining of CRC tissues was also performed. Brown/pink color = SATB2 or β-catenin. IgG was used as a negative control (data not shown). H & E staining of the same tissue was also performed. Representative photographs of 40 CRC samples. (**G**) Protein expression of SATB2, β-catenin, cyclin D1 and Survivin. Cell lysates from CSC/Scrambled and CSCs/SATB2 shRNA groups were prepared and the expression of SATB2, β-catenin, cyclin D1 and Survivin was measured by the WB analysis. (**H**) TCF/LEF transcriptional activity in CRC CSC/Scrambled and CSCs/SATB2 shRNA groups was measured by luciferase assay. Data represent mean (n = 4) ±SD. *Significantly different from scrambled control group (P < 0.05).

Since SATB2 transformed cells gain the phenotype of CSCs, we next examined the effects of inhibiting SATB2 by shRNA on the markers of CSCs, pluripotency/self-renewal, cell cycle, cell survival/proliferation. Transduction of colon CSCs with lentiviral particles expressing SATB2 shRNA inhibited the expression SATB2 (Fig. 7A). SATB2 shRNA inhibited the mRNA expression of stem cell markers (CD133 and CD44) and pluripotency maintaining factors (c-Myc and Nanog) (Fig. 7B and C). We further confirmed these data with the Western blot analysis. SATB2 shRNA inhibited the protein expression of CD44, CD133, c-Myc and Nanog in CRC CSCs (Fig. 7B and C).

We next examined whether inhibition of SATB2 regulates Cyclin D1, XIAP and Bcl-2 expression, and genes which regulate cell cycle, survival and proliferation. As shown in Fig. 7D, SATB2 shRNA inhibited the expression of Cyclin D1, XIAP and Bcl-2 in colon CSCs at mRNA and protein levels. These data suggest that SATB2 can regulate the expression of stem cell markers, pluripotency maintaining factors, cell cycle and cell survival/proliferation in colorectal CSCs.

Since spheroid formation is one of the characteristics of CSCs, we next examined whether inhibition of SATB2 expression by shRNA deteriorates the ability of CSCs’s to form spheroids. As shown in Fig. 7E, CSCs/SATB2 shRNA groups formed significantly smaller spheroids consisting of fewer than 20 cells, whereas CSCs/Scrambled cells form larger spheroids with thousands of cells. These data suggested that SATB2 is essential for CSC characteristics.

**Figure 8:**
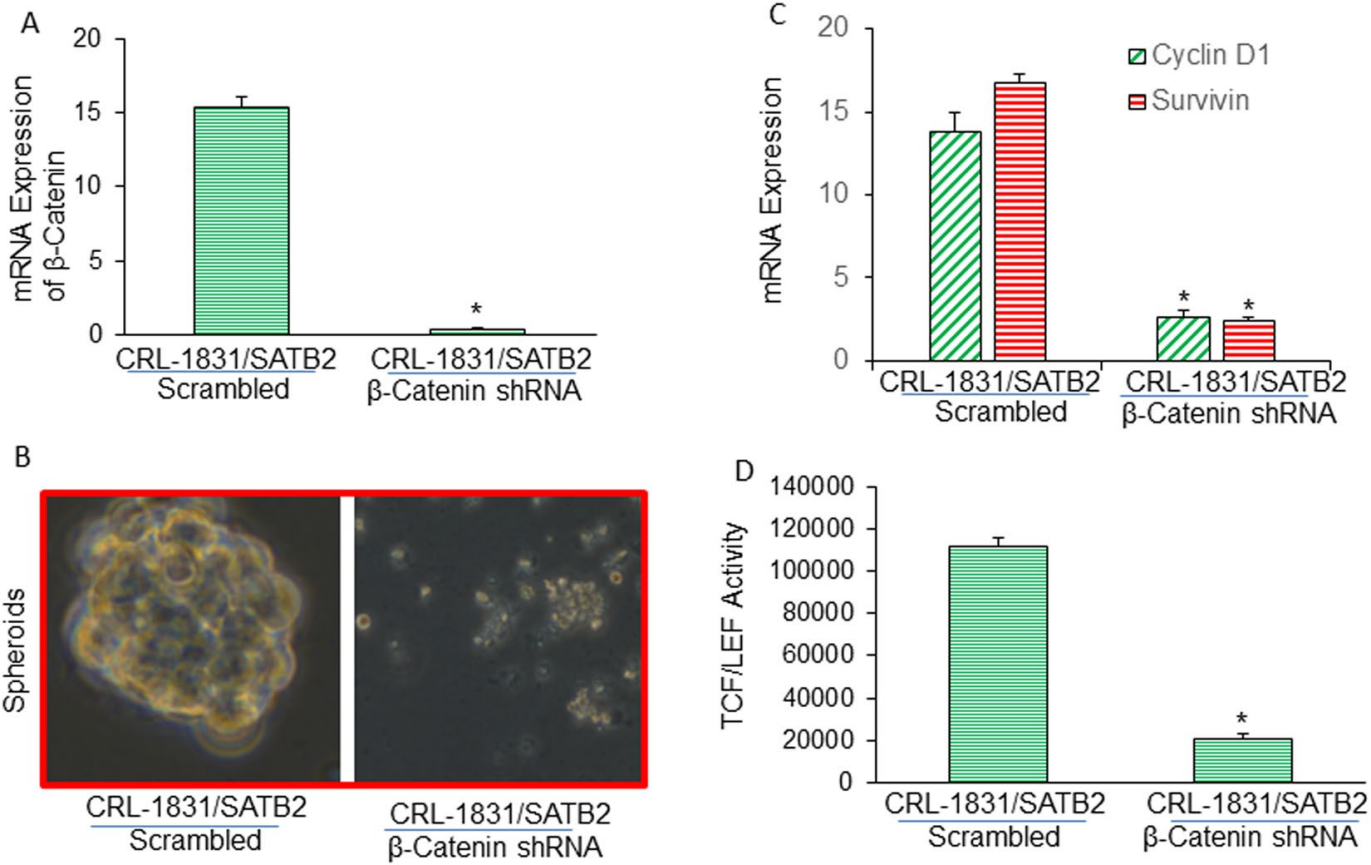
Suppression of β-catenin blocks SATB2-induced spheroid formation, Cyclin D1 and Survivin expression, and TCF/LEF activity. (**A**), CRL-1831/SATB2 cells (SATB2 overexpressing CRL-1831 cells) were transduced with lentiviral particles expressing either scrambled or β-catenin shRNA. β-catenin mRNA expression was measured by qRT-PCR. Data represent mean (n = 4) ±SD. *Significantly different between groups (P < 0.05). (**B**) Suppression of β-catenin blocks SATB2-induced spheroid formation. CRL-1831/SATB2 cells were transduced with lentiviral particles expressing either scrambled or β-catenin shRNA (pLKO.1 puro shRNA β-catenin). Cells were grown in suspension, and spheroids formed after 2 weeks were photographed. (**C**) Suppression of β-catenin inhibits Cyclin D1 and Survivin expression in CRL-1831/SATB2 cells. CRL-1831/SATB2 cells were transduced with lentiviral particles expressing either scrambled or β-catenin shRNA. Cyclin D1 and Survivin expression was measured by qRT-PCR. Data represent mean (n = 4) ±SD. *Significantly different between groups (P < 0.05). (**D**) Suppression of β-catenin inhibits TCF/LEF activity in CRL-1831/SATB2 cells. CRL-1831/SATB2 cells were transduced with lentiviral particles expressing either scrambled or β-catenin shRNA. TCF/LEF activity was measured by luciferase assay. Data represent mean (n = 4) ±SD. *Significantly different from scrambled control group (P < 0.05).

The role of β-catenin/TCF-LEF pathway in generation of CSCs and CRC carcinogenesis is well documented^46–48^. We first assessed whether there is any correlation in the expression of SATB2 and β-catenin in human CRC tissues. IHC data demonstrated that SATB2 expression is positively correlated with β-catenin expression (Fig. 7F). We next examined whether β-catenin/TCF-LEF pathway mediates the effects of SATB2 in CSCs by measuring the expression of β-catenin and TCF-LEF targets, and TCF-LEF transcriptional activity. SATB2 shRNA inhibited the expression of SATB2, and β-catenin expression, and TCF/LEF targets cyclin D1 and Survivin (Fig. 7G). Furthermore, inhibition of SATB2 by shRNA attenuated TCF-LEF transcriptional activity (Fig. 7H). In another approach, we silenced the expression of β-catenin in SATB2-transformed CRL-1831 cells (which gained the phenotypes of stem cells) to examine the contribution of Wnt/TCF-LEF pathway in SATB2-mediated transformation (Fig. 8). As shown in Fig. 8A, β-catenin shRNA inhibited the expression of β-catenin as measured by qRT-PCR. We next examined whether β-catenin shRNA can inhibit the ability of CRL-1831/SATB2 cells to form spheroids in suspension and suppress the expression of TCF/LEF targets. Silencing of β-catenin inhibited spheroid formation in suspension and the expression of Cyclin D1 and Survivin in CRL-1831/SATB2 cells (Fig. 8B and C). Furthermore, silencing of β-catenin by shRNA inhibited TCF-LEF activity in CRL-1831/SATB2 cells (Fig. 8D). Overall, our data demonstrate that β-catenin/TCF-LEF pathway mediates the biological effects of SATB2.

## Discussion

We have demonstrated for the first time that SATB2 can induce malignant transformation in normal colorectal epithelium by converting cells which are capable of expressing stem cell markers and pluripotency maintaining factor. The transformed CRL-1831 cells appear to be genotypically and phenotypically similar to CSCs/progenitor cells found in the human colorectal cancer. Since SATB2 is not expressed in normal CRL-1831 cells, but it is highly expressed in human CRC cell lines, and primary cancer tissues, it can be used as a diagnostic and prognostic biomarker of CRC. Furthermore, inhibition of SATB2 in CRC cells suppresses cell proliferation, colony formation, cell motility, and the expression of Slug, Snail, and Zeb1 (EMT-related transcription factors), and N-cadherin. Inhibition of SATB2 expression in CRC CSCs also abrogated CSC characteristics by inhibiting the expression of c-Myc, Nanog, Bcl-2, XIAP, cyclin D1, CD133 and CD44. Since the SATB2 protein is highly expressed in the CRC cell lines and tissues, it can be an attractive target for therapy, diagnosis and prognosis.

Cellular transformation is the first characteristic feature that epithelial cells gain during cancer development. During transformation, the most abundant changes occur in cellular morphology, growth, and metabolism^49–51^. SATB2 is a DNA-binding protein that specifically binds nuclear matrix attachment regions and plays role in transcriptional regulation and chromatin remodeling^16, 52, 53^. However, the role of SATB2 in malignant transformation of colorectal epithelium has not been demonstrated. The CSCs/tumor initiating cells are believed to be the cause of cancer initiation, metastasis and resistance to therapy^8, 9, 54^. We have demonstrated that human normal colon epithelial CRL-1831 cells do not express SATB2; however it is highly expressed in CRC cell lines, CSCs and primary tumors. Overexpression of SATB2 in CRL-1831 cells induces cellular transformation, and transformed CRL-1831 cells gain the phenotypes of CSCs as they express stem cell markers and pluripotency maintaining factors. SATB2 in combination with CK20 can identify more than 95% of all colorectal carcinoma^19^. A recent study using 50 paired CRC and normal tissues has observed an increased expression of SATB2 in CRC tissues compared to normal tissues^55^. In contrary, downregulation of SATB2 expression was shown to be associated with enhanced cell growth, metastasis and poor prognosis in CRC^56, 57^ and overexpression of SATB2 repressed cancer cell proliferation, migration and invasion by inhibiting ERK5^58^. Similar to the findings in this paper, using breast cancer model we and others have demonstrated that silencing SATB2 expression significantly inhibited cell proliferation, migration and invasion of cancer cell lines and CSCs^59, 60^. Jiang *et al*.^61^ demonstrated the upregulation of SATB2 in HCC (hepatocellular carcinoma) cancer tissues and cell lines. Furthermore, in another study we have recently demonstrated that SATB2 induced transformation of pancreatic ductal epithelial cells which gained the phenotypes of CSCs/progenitor cells with induced expression of stem cell transcription factors^62^. It suggests that SATB2 is capable of inducing transformation, and it may be a driver of those down-stream genes which are involved in generating progenitor cells/CSCs. Based on these studies, it appears that the expression of SATB2 is regulated by differentiation stage of the CRC tissues.

Recent studies have identified certain microRNAs (miRNAs) which regulated cancer cell progression, and metastasis by modulating the expression of SATB2. miRNA-211 and miR-449a inhibited HCC and CRC progression by down-regulating SATB2 expression, and overexpression of SATB2 counteracted the inhibitory effects of these miRNAs on cell proliferation and tumor growth, suggesting the oncogenic potential of SATB2^55, 61^. In contrast, miRNA-182 and miRNA-31 promoted CRC proliferation, EMT and metastasis by suppressing SATB2^63, 64^. Based on these studies it is not clear why certain miRNAs would promote or inhibit cancer progression and metastasis by suppressing SATB2. The reasons for these differences may be due to fact that miRNAs target multiple genes with diverse cellular functions at the same time.

EMT is a process via which epithelial cells undergo morphological changes to a motile mesenchymal phenotype, a phenomenon implicated in tumor progression and metastasis^41^. The cells undergoing EMT upregulate the expression of cell motility-related proteins and show increased migration and invasion^41^. Therefore, prevention of EMT could be an important event in inhibiting tumor metastasis. The mesenchymal-to-epithelial transition (MET) is an intrinsically mechanical process consisting of a multi-step progression where autonomous mesenchymal cells tightly linked with each other, leading to polarization of epithelial cells. METs are also essential in formation of distant/secondary metastasis. Our data demonstrate that SATB2 overexpression promotes cell motility, migration and invasion by modulating EMT-related genes and transcription factors. In contrast, SATB2 inhibition by shRNA reversed EMT characteristics. Our studies suggest that SATB2 can promote cancer cell EMT and also metastasis.

The SATB2 binding sites are present in β-catenin promoter. Our data showed that the expression of SATB2 protein positively correlated with nuclear β-catenin expression. Furthermore, inhibition of SATB2 by shRNA attenuated β-catenin expression and TCF-LEF transcriptional activity. In addition, SATB2 shRNA inhibited the expression of TCF-LEF target genes CD44, c-Myc, Nanog, Cyclin D1, XIAP, Survivin and Bcl-2 in colorectal CSCs. Similarly other studies have demonstrated the regulation of stem cell markers, pluripotency maintaining factors and cell survival, and cell cycle genes by β-catenin-TCF/LEF pathway^65–70^. These data suggest that SATB2 can regulate pluripotency and self-renewal of colorectal CSCs through β-catenin/TCF-LEF pathway.

In conclusion, our data demonstrate that overexpression of SATB2 induces CRL-1831 cell transformation, and these transformed cells possess properties of CSCs/progenitor cells. The transformed CRL-1831 cells appear to be genotypically and phenotypically similar to CSCs/progenitor cells found in the human CRC. The expression of SATB2 was positively correlated with β-catenin expression in CRC tissues and CSCs. Inhibition of SATB2 in CSCs suppresses cell proliferation, colony formation, spheroid formation and EMT. Finally, our data suggest that β-catenin/TCF-LEF pathway may mediate the biological effects of SATB2 in CRC. These studies support the role of SATB2 in transformation and dedifferentiation, and suggest its oncogenic role in CRC initiation, progression and metastasis. Future studies in transgenic mice are needed to confirm these findings of SATB2 in CRC.
